# The Gut Commensal Microbiome of *Drosophila melanogaster* Is Modified by the Endosymbiont *Wolbachia*

**DOI:** 10.1128/mSphere.00287-17

**Published:** 2017-09-13

**Authors:** Rama K. Simhadri, Eva M. Fast, Rong Guo, Michaela J. Schultz, Natalie Vaisman, Luis Ortiz, Joanna Bybee, Barton E. Slatko, Horacio M. Frydman

**Affiliations:** a Department of Biology, Boston University, Boston, Massachusetts, USA; b NEIDL (National Emerging Infectious Diseases Laboratory), Boston University, Boston, Massachusetts, USA; c Genome Biology Division, New England Biolabs, Inc., Ipswich, Massachusetts, USA; Loyola University Chicago

**Keywords:** *Drosophila* microbiome, gut microbiome, symbiosis, *Wolbachia*

## Abstract

Endosymbiotic *Wolbachia* bacteria and the gut microbiome have independently been shown to affect several aspects of insect biology, including reproduction, development, life span, stem cell activity, and resistance to human pathogens, in insect vectors. This work shows that *Wolbachia* bacteria, which reside mainly in the fly germline, affect the microbial species present in the fly gut in a lab-reared strain. *Drosophila melanogaster* hosts two main genera of commensal bacteria—*Acetobacter* and *Lactobacillus*. *Wolbachia*-infected flies have significantly reduced titers of *Acetobacter*. Sampling of the microbiome of axenic flies fed with equal proportions of both bacteria shows that the presence of *Wolbachia* bacteria is a significant determinant of the composition of the microbiome throughout fly development. However, this effect is host genotype dependent. To investigate the mechanism of microbiome modulation, the effect of *Wolbachia* bacteria on Imd and reactive oxygen species pathways, the main regulators of immune response in the fly gut, was measured. The presence of *Wolbachia* bacteria does not induce significant changes in the expression of the genes for the effector molecules in either pathway. Furthermore, microbiome modulation is not due to direct interaction between *Wolbachia* bacteria and gut microbes. Confocal analysis shows that *Wolbachia* bacteria are absent from the gut lumen. These results indicate that the mechanistic basis of the modulation of composition of the microbiome by *Wolbachia* bacteria is more complex than a direct bacterial interaction or the effect of *Wolbachia* bacteria on fly immunity. The findings reported here highlight the importance of considering the composition of the gut microbiome and host genetic background during *Wolbachia*-induced phenotypic studies and when formulating microbe-based disease vector control strategies.

**IMPORTANCE** *Wolbachia* bacteria are intracellular bacteria present in the microbiome of a large fraction of insects and parasitic nematodes. They can block mosquitos’ ability to transmit several infectious disease-causing pathogens, including Zika, dengue, chikungunya, and West Nile viruses and malaria parasites. Certain extracellular bacteria present in the gut lumen of these insects can also block pathogen transmission. However, our understanding of interactions between *Wolbachia* and gut bacteria and how they influence each other is limited. Here we show that the presence of *Wolbachia* strain *w*Mel changes the composition of gut commensal bacteria in the fruit fly. Our findings implicate interactions between bacterial species as a key factor in determining the overall composition of the microbiome and thus reveal new paradigms to consider in the development of disease control strategies.

## Abstract (precis)

*Wolbachia* bacteria are intracellular bacteria present in the microbiome of a large fraction of insects and parasitic nematodes. They can block mosquitos’ ability to transmit several infectious disease-causing pathogens, including Zika, dengue, chikungunya, and West Nile viruses and malaria parasites. Certain extracellular bacteria present in the gut lumen of these insects can also block pathogen transmission. However, our understanding of interactions between *Wolbachia* and gut bacteria and how they influence each other is limited. Here we show that the presence of *Wolbachia* strain *w*Mel changes the composition of gut commensal bacteria in the fruit fly. Our findings implicate interactions between bacterial species as a key factor in determining the overall composition of the microbiome and thus reveal new paradigms to consider in the development of disease control strategies.

## INTRODUCTION

All animals host a diverse and extensive microbial community, referred to as the microbiome. The microbiome plays a pivotal role in host development and growth (1). Insects provide a prime example of the relevance of microbes in the host, having a major impact on several aspects of host biology, including physiology, immunity, and evolution (2, 3). Microbes also play an important role in the reproductive capabilities of the insect host. In some cases, they hijack the insect’s cellular pathways to alter fecundity, sometimes evolving to become essential symbionts for host reproductive success (reviewed in references 2 and 4).

These reproductive symbionts can either be transmitted vertically by the parents or acquired horizontally from the environment. *Wolbachia* bacteria are obligate endosymbionts that are found in about 40% of all insect species and cause various types of reproductive phenotypes that generally favor their vertical transmission and spread in populations (4–6). Apart from reproductive manipulation, *Wolbachia* bacteria are also known to cause a plethora of other changes in their hosts, such as altering insulin signaling (7) and providing resistance to certain pathogens (reviewed in reference 8).

While *Wolbachia* bacteria usually infect reproductive tissues, other microbes infecting different tissues coexist in the insect host. The repertoire of the microbes associated with the host can vary on the basis of both environmental factors (9–12) and the host genotype (13). In *Drosophila*, the diversity of the microbiome is very low and depends greatly on the host diet, and lab-reared flies have comparatively fewer taxa than wild-caught flies (10). *Acetobacter* and *Lactobacillus* are the most commonly found genera of bacteria in *Drosophila melanogaster*, both in lab-reared flies and in nature (14, 15). They affect development, metabolism, and behavior (reviewed in reference 14).

Recent studies have explored the effects of various microbes on the composition of the insect microbiome, implicating interactions between microbes to play a significant role in the determination of the composition of the microbiome (16, 17). In particular, Hughes et al. showed that the presence of a specific bacterium, *Asaia* sp., prevents the stable transmission and maintenance of *Wolbachia* bacteria in the host. Upon antibiotic treatment and removal of *Asaia* bacteria, the mosquito host is able to successfully carry and vertically transmit *Wolbachia* bacteria.

Here we address the converse question of whether *Wolbachia* bacteria have a role in the determination of the composition of the microbiome. Sequencing-based approaches to the identification of the microbiome of *Wolbachia-*infected strains have been complicated because *Wolbachia* bacteria have been shown to be overrepresented in the microbiome of field-collected insects and valuable information about the composition of other microbes is lost. We solved this issue by restriction digestion of the *Wolbachia* 16S rRNA gene prior to profiling the composition of the microbiome. We then found that the presence of *Wolbachia* bacteria induces significant changes in the host microbiome. These changes persist throughout development in a stable lab stock. On further investigation with gnotobiotic organisms, we found that the *Wolbachia*-induced effect is independent of vertical transmission of the composition of the microbiome. The host genetic background contributes to the interaction of *Wolbachia* bacteria with the microbiota. Furthermore, transcriptional profiling of immune effector molecules controlled by the Imd and JAK/STAT pathways suggests that the mechanism of this *Wolbachia*-driven change in the gut microbiome is independent of classical host immunity pathways.

## RESULTS

**FIG 1:**
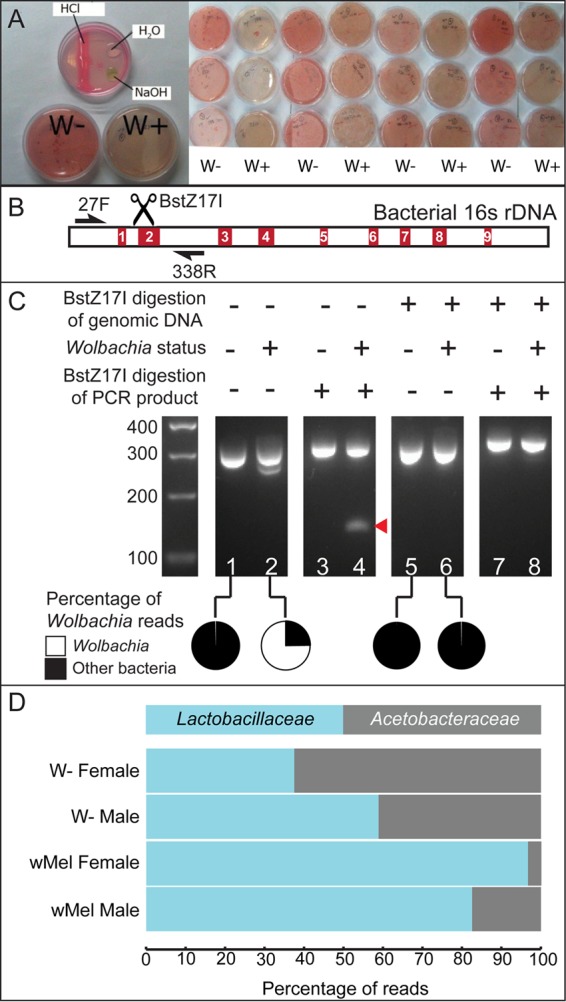
16S rRNA gene profiling of *D. melanogaster* shows reduction of *Acetobacteraceae* levels in *Wolbachia*-infected adult flies. (A) Grape juice agar plates used for *D. mauritiana* egg collection. Grape juice acts as a pH indicator, turning pink under acidic conditions and yellow under basic conditions. Over time, the microbial composition of the feces of *Wolbachia*-infected flies turns plates more yellowish than plates used by *Wolbachia-*free flies. (B) Schematic of the bacterial 16S rRNA gene. Hypervariable regions V1 to V9 are red. Primers (arrows, 27F and 338R) amplify regions V1 and V2. In BstZ17I restriction enzyme digestion of the total 16S rRNA gene pool, only the *Wolbachia* rRNA gene between V1 and V2 is selectively digested. (C) Agarose gel image showing the 16S rRNA gene PCR products from the microbiome of *D. melanogaster* and efficient digestion of the *Wolbachia* 16S rRNA gene amplicon by the BstZ17I restriction enzyme. The red arrowhead indicates the digested *Wolbachia* product. The pie charts indicate the percentages of *Wolbachia* reads before and after BstZ17I digestion. (D) 16S rRNA gene profiles of male and female *w*Mel-free (W−) or -infected *D. melanogaster*. The proportions of *Acetobacteraceae* are significantly different under every pair of conditions (*P* < 0.0001, chi-square test with Yates correction).

Previous study in our lab showed that *Wolbachia* bacteria affect the fecundity of *Drosophila mauritiana* flies (6). In a parallel study, we observed that the presence of *Wolbachia* bacteria in *D. mauritiana* changed the color of grape juice plates over time. The plates used for egg collection (see Materials and Methods) from *Wolbachia-*free flies were pink but displayed a yellowish tinge when exposed to *Wolbachia*-infected flies (Fig. 1A). Adding an acid or a base to the grape juice plates indicated that the color change is pH dependent (Fig. 1A). Since flies defecate on the agar medium, we suspected that the fecal microbiota-derived metabolites might contribute toward determining the final pH of the agar. In addition, if there is a difference in microbial composition between *Wolbachia-*free and -infected flies, this might contribute to the differential coloration of the grape juice agar plates. Since the *D. mauritiana* system does not have powerful genetic tools with which to further analyze this phenotype, we investigated the possibility that *Wolbachia* bacteria have an effect on the microbiome of *D. melanogaster* flies. Results obtained with *D. melanogaster* will be more broadly applicable than those obtained with *D. mauritiana*.

For this analysis, we chose to examine *D. melanogaster* upd-Gal4; Cyo/Sco; P(UAS-hPF)B, here referred to as upd>hPABP-Flag flies. The hPABP-Flag transgene allows the isolation of mRNA from specific cell types for transcriptional profiling (18). We used the upd promoter, which is active in the stem cell niche (hub) of the testis, to drive the expression of hPABP-Flag. Since *Wolbachia* bacteria show tropism to the hubs in males (19), this construct allows for the isolation of RNA for studying gene expression patterns that are altered because of *Wolbachia* infection. The upd>hPABP-Flag strain was isogenized and used for molecular characterization of the effects of *Wolbachia* bacteria on stem cells and their niches (see Materials and Methods). Since the microbiome has been shown to affect host phenotypes such as fecundity (20, 21), which is a direct result of stem cell activity (6), we considered testing of the effects of *Wolbachia* bacteria on the microbiome to be important especially in this specific genotype.

### *Wolbachia* 16S rRNA gene sequences can be efficiently removed prior to sequencing.

To determine the microbial content of upd>hPABP-Flag flies, we used high-throughput sequencing of the 16S rRNA gene amplicons from total genomic DNA isolated from flies. A major hurdle in the sequencing of 16S rRNA gene sequences from flies infected with *Wolbachia* bacteria is the overrepresentation of *Wolbachia* sequences (10). BstZ17I digestion of the total genomic DNA specifically prevents amplification of the *Wolbachia* 16S rRNA gene, as the restriction enzyme cleaves between the V1 and V2 regions. Microbes normally found in *Drosophila* flies do not contain the BstZ17I restriction sites. Other bacteria containing this site are present in the orders *Rhizobiales* and *Myxococcales* and non-*Wolbachia* members of the order *Rickettsiales* that have been reported to be absent or occur at very low numbers in *Drosophila* flies (22). We found that BstZ17I digestion prior to 16S rRNA gene PCR of the V1 and V2 regions effectively removed most of the *Wolbachia* amplicons (Fig. 1B and C; see Table S1 in the supplemental material). While more than 70% of the reads originated from *Wolbachia* bacteria in the case of undigested genomic DNA, the BstZ7I-digested genomic DNA produced less than 1% *Wolbachia* reads (Fig. 1C, lane 6). We also confirmed the *Wolbachia* strain present to be *w*Mel by variable-number tandem-repeat (VNTR) analysis (Fig. S1).

10.1128/mSphere.00287-17.1 FIG S1  VNTR analysis to determine the *Wolbachia* strain. Different *Wolbachia* strains have different numbers of repeats in some VNTRs, specifically, 105 and 141. The expected sizes in *w*Mel are 1,347 bp for VNTR 105 and 1,330 bp for VNTR 141; in *w*MelCS, they are 1,241 bp for VNTR 105 and 1,189 bp for VNTR 141 (67). Lanes 1 and 2 in both gels are the *Wolbachia-*free and *w*Mel-infected flies used in this study, respectively. Lanes 3 are flies infected with *w*MelCS (not used in this study) for comparison. Download FIG S1, TIF file, 0.2 MB. Copyright © 2017 Simhadri et al. 2017 Simhadri et al. This content is distributed under the terms of the Creative Commons Attribution 4.0 International license.

10.1128/mSphere.00287-17.7 TABLE S1  Number of reads for each sample and effectiveness of Bstz17I digestion. Sequencing of the 16S rRNA gene PCR products of either BstZ17I-digested or undigested total genomic DNA of flies shows that the BstZ17I enzyme is capable of eliminating the amplification of the *Wolbachia* 16S rRNA gene. Download TABLE S1, DOCX file, 0.01 MB. Copyright © 2017 Simhadri et al. 2017 Simhadri et al. This content is distributed under the terms of the Creative Commons Attribution 4.0 International license.

10.1128/mSphere.00287-17.8 TABLE S2  Primers used for amplification and sequencing. The different regions of the primers are Illumina adaptor (red), indexing barcode (black), primer pad and linker (green), and 16S rRNA gene annealing primer (blue) sequences. Download TABLE S2, DOCX file, 0.01 MB. Copyright © 2017 Simhadri et al. 2017 Simhadri et al. This content is distributed under the terms of the Creative Commons Attribution 4.0 International license.

### *Wolbachia* bacteria reduce the proportion of *Acetobacteraceae.*

To assess the effect of *Wolbachia* on bacterial composition associated with these flies, we sequenced the non-*Wolbachia* 16S rRNA gene from 0- to 2-week-old adult male and female flies separately (*n* = 5 flies per sample, 20 flies total). We then grouped the 16S rRNA gene sequences into 97% identical operational taxonomic units. We found that the vast majority (>99.8%) of the microbiome of both *Wolbachia-*free and -infected flies is restricted to only two families—*Acetobacteraceae* and *Lactobacillaceae* (Fig. 1D; Table S3). Intriguingly, there was a striking contrast between the proportions of the two families of bacteria in *Wolbachia-*free and -infected flies. Members of the family *Acetobacteraceae* make up less than 20% (17% in males and 3% in females) of the microbes in *w*Mel-infected flies and more than 40% (41% in males and 62% in females) of those in *Wolbachia-*free flies (Fig. 1D).

10.1128/mSphere.00287-17.9 TABLE S3  Percentages of (non-*Wolbachia*) bacterial taxa found in each of the samples by 16S rRNA gene sequencing. The two most abundant families of bacteria are *Acetobacteraceae* and *Lactobacillaceae*, which constitute more than 99.8% of the reads in any sample. Download TABLE S3, DOCX file, 0.01 MB. Copyright © 2017 Simhadri et al. 2017 Simhadri et al. This content is distributed under the terms of the Creative Commons Attribution 4.0 International license.

To determine the specific bacterial species present in these flies, we used culture-based techniques, followed by Sanger sequencing of the 16S rRNA gene of the bacterial isolates and whole-genome sequencing with the PacBio sequencing platform (see Materials and Methods). Consistent with our 16S rRNA sequencing data, we found that *Acetobacter pasteurianus* and *Lactobacillus plantarum* are the only two species residing in this fly strain. We then designed species-specific primers against their *glmS* gene for further analysis with PCR-based assays (see Fig. S2 and Materials and Methods).

10.1128/mSphere.00287-17.2 FIG S2  Species-specific primers designed for qPCR. Primers were designed against the *glmS* gene of both species. The PCR results show that the primers are specific, having no cross-species targets. *A*. *p* is *A. pasteurianus*, and *L*. *p* is *L. plantarum*. Download FIG S2, TIF file, 0.1 MB. Copyright © 2017 Simhadri et al. 2017 Simhadri et al. This content is distributed under the terms of the Creative Commons Attribution 4.0 International license.

### *Wolbachia*-induced reduction of *A. pasteurianus* levels persists throughout development.

**FIG 2:**
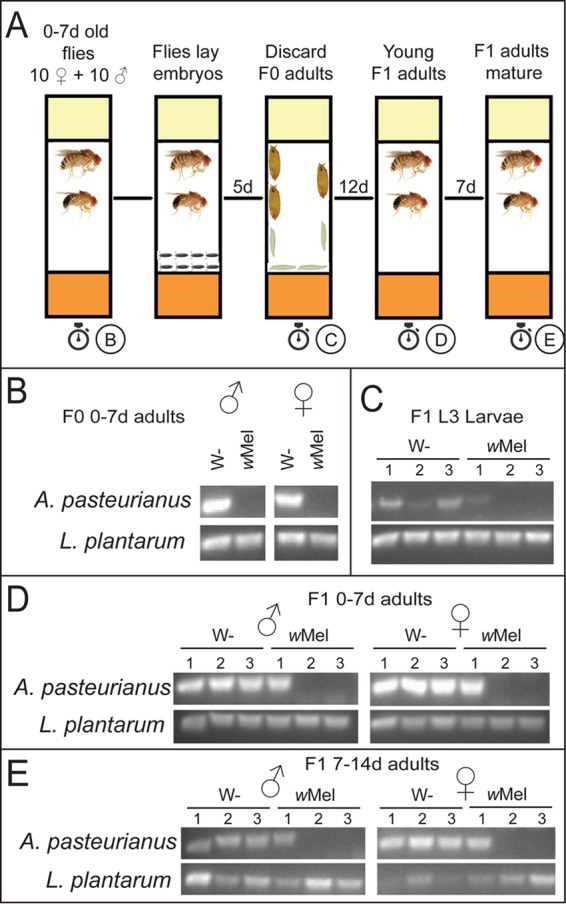
*Wolbachia* bacteria suppress *A. pasteurianus* across various life stages of *D. melanogaster*. (A) Schematic of the experimental setup used. Stopwatches indicate sample collection times. (B to E) PCR products obtained with *A. pasteurianus* and *L. plantarum* species-specific primers from BstZ17I-digested total genomic DNA from *w*Mel-free (W−) or -infected *D. melanogaster*. (B) The parental flies are F0 0- to 7-day-old males and females. (C to E) Experiments were done in triplicate, and each sample consisted of five adults (B, D, E) or five larvae (C). Vial numbers are above the gel lanes. (C) F1 unsexed L3 larvae (*n* = 3), (D) F1 0- to 7-day-old male and female flies (*n* = 3). (E) F1 7- to 14-day-old male and female flies (*n* = 3).

To further corroborate the differences in the composition of the microbiome shown by the deep sequencing results, we performed PCR of *Wolbachia-*free and -infected *Drosophila* stocks with species-specific primers. We also analyzed the effect of *Wolbachia* bacteria on the microbiome during development (see the diagram of the experiment in Fig. 2A); 35 adult males and 35 adult females were used as the parental generation for the experiment. To analyze the composition of the parental microbiota, five males and females each were surface sterilized and DNA was extracted. The remaining 30 males and females each were split into three replicates of 10 male and 10 female parents for both the *w*Mel*-*free and -infected flies. After 7 days of egg laying, these F0 adults were removed from the vials. Five individuals each from the next generation (F1) were collected in triplicate at multiple life stages—L3 larvae, 0- to 7-day-old adults, and 7- to 14-day-old adults. DNA was extracted from surface-sterilized organisms and digested with BstZ17I to exclude *Wolbachia* 16S rRNA gene amplification. On performing PCR with species-specific primers against the two isolated species, we found that *A. pasteurianus* was not detected in the majority of the *Wolbachia*-infected samples. There was no detectable *A. pasteurianus* in the parent flies, two out of three replicates of the F1 L3 larvae, and both the adult stages. That is, in 12 out of a total of 17 *Wolbachia*-infected samples, *A. pasteurianus* was absent, compared to its detection in 17 out of 17 *Wolbachia-*free flies. The lack of *A. pasteurianus* was observed in all of the life stages sampled (Fig. 2B to E). In the samples that did have *A. pasteurianus*, quantification by quantitative PCR (qPCR) showed that the levels of the bacteria were about 10-fold lower in the *Wolbachia*-infected organisms than in their *Wolbachia-*free counterparts (Fig. S3). These results confirm that the presence of *Wolbachia* bacteria consistently reduces the levels of *A. pasteurianus*.

10.1128/mSphere.00287-17.3 FIG S3  Quantification of effects of *Wolbachia* bacteria on *A. pasteurianus* levels in flies during development. (A to C) Relative levels of *A. pasteurianus* and *L. plantarum* in *w*Mel-infected flies and those in *Wolbachia-*free flies. (A) F1 unsexed L3 larvae. (B) F1 0- to 7-day-old male and female flies. (C) F1 7- to 14-day-old male and female flies. (A to C) *A. pasteurianus* was absent from two out of the three replicates of *Wolbachia*-infected vials tested, hence the absence of error bars in the *A. pasteurianus* bars. Bar graphs show mean values (*n* = 3 when the qPCR produced amplicons), and error bars show standard deviations. Download FIG S3, TIF file, 0.6 MB. Copyright © 2017 Simhadri et al. 2017 Simhadri et al. This content is distributed under the terms of the Creative Commons Attribution 4.0 International license.

### *Wolbachia* bacteria also reduce *A. pasteurianus* levels in gnotobiotic organisms.

**FIG 3:**
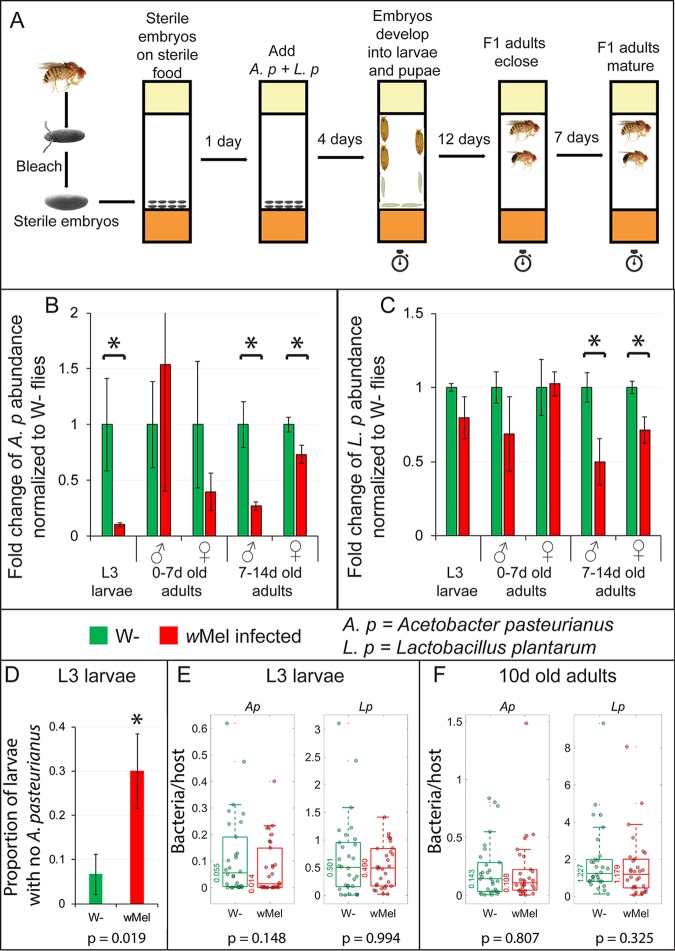
*Wolbachia* infection reduces *A. pasteurianus* levels in L3 larvae of gnotobiotic flies. (A) Schematic of the experimental setup used. Stopwatches indicate sample collection times. (B, C) qPCR of *A. pasteurianus* (B) and *L. plantarum* (C) titers in *w*Mel-infected gnotobiotic flies compared to those in *Wolbachia*-free (W−) gnotobiotic flies, normalized to 16S rRNA gene levels, in L3 larvae, 0- to 7-day-old adults, and 7- to 14-day-old adults. Bar graphs show mean values (three biological replicates of a sample of five individuals per replicate), and error bars show standard deviations. Asterisks indicate statistically significant differences (*P* < 0.05, Student *t* test). (D) Proportion of individual L3 larvae that had no *A. pasteurianus* in the gut (*n* = 30, chi-square test; error bars show confidence intervals). (E, F) Box-and-whisker plots of levels of bacteria in L3 larvae (E) and 10-day-old adults (F) (*n* = 30). Median values are shown next to the boxes, and *P* values (two-sided Wilcoxon rank sum test) are reported.

Besides the presence of *Wolbachia* bacteria, another factor that could influence the microbiome is the difference in the relative abundance of each bacterium passed on by the parents. The general absence of *A. pasteurianus* in the F1 generation (Fig. 2) could have been due to the low abundance of this bacterium in the parent flies and not necessarily caused by *Wolbachia* infection. To test this possibility, we eliminated the variability in the quantities of the bacterial species imparted by the parent flies between the various samples. We produced gnotobiotic organisms that were infected with equal quantities of *A. pasteurianus* and *L. plantarum* (Fig. 3A). Ten-microliter volumes of bleach-sterilized eggs of *w*Mel*-*free and -infected flies were seeded on sterile fly food in triplicate. To minimize growth rate and survival differences between the bacteria in the fly food, 1,000 CFU of each of the bacteria were added to the food after a majority of the eggs hatched (see Materials and Methods). Five individuals each from various life stages—L3 larvae, 0- to 7-day-old adults, and 7- to 14-day-old adults—were then collected from each biological replicate. Upon performing qPCR for each of the bacteria, we found that the levels of *A. pasteurianus* were indeed about 10-fold higher (*P* < 0.05) in the *Wolbachia*-free larvae than in the *w*Mel-infected larvae (Fig. 3B). However, the 0- to 7-day-old adults, either *Wolbachia* free or infected, had no statistically significant difference in the levels of *A. pasteurianus* (Fig. 3B). Finally, the 7- to 14-day-old *w*Mel-infected adults had lower levels of both *A. pasteurianus* and *L. plantarum* than the *Wolbachia-*free flies (Fig. 3B and C).

To elucidate if this reduction of *A. pasteurianus* levels is due to an overall reduction of the bacteria in all *Wolbachia*-infected flies or to the complete lack of *A. pasteurianus* in large fractions of *Wolbachia*-infected flies, we sampled individual organisms to assay the levels of each gut bacterium relative to the host. Gnotobiotic organisms were produced as described above. Three separate experiments were performed, and 10 individuals (L3 larvae and 10-day-old adults) were sampled from each experiment. On performing qPCRs with species-specific primers and comparing the results to the host DNA, we found that about 30% of the *Wolbachia*-infected L3 larvae did not harbor any *A. pasteurianus*, compared to 6% of *Wolbachia*-free flies (*P* = 0.019) (Fig. 3D). Further, the median levels of *A. pasteurianus* in *Wolbachia*-infected flies were 4-fold lower than in *Wolbachia*-free flies (*P* = 0.148), while the levels of *L. plantarum* were unaffected by *Wolbachia* bacteria (Fig. 3E). In the 10-day-old adult flies, neither *A. pasteurianus* nor *L. plantarum* levels relative to the host were affected by the presence of *Wolbachia* bacteria (Fig. 3F). To ensure that this was not an artifact of a lack of *A. pasteurianus* in the food, we sampled the bacterial levels in the food (see Materials and Methods), and the levels of both bacteria were comparable in bottles of *Wolbachia-*free and -infected L3 larvae and 10-day-old adults (Fig. S4).

10.1128/mSphere.00287-17.4 FIG S4  Bacterial levels in the food of upd>hPABP-Flag flies. Relative levels of each bacterial species per nanogram of DNA obtained from food, normalized to the levels in food inhabited by *Wolbachia-*free flies are shown. Bar graphs represent the mean values of three experiments, and error bars show standard deviations. *P* values were determined by Student *t* tests. Download FIG S4, TIF file, 0.2 MB. Copyright © 2017 Simhadri et al. 2017 Simhadri et al. This content is distributed under the terms of the Creative Commons Attribution 4.0 International license.

From these results, the greatest species-specific effect of *Wolbachia* bacteria on the microbiome appears to occur at an earlier developmental stage but, importantly, is independent of the transmission of a specific microbial composition from the parents.

### *Wolbachia*-related microbiome changes are host genotype dependent.

We tested another, unrelated, genotype to assess if *Wolbachia*-induced microbiome changes are sensitive to the host genotype. We used isogenized white homozygous flies (see Materials and Methods) and performed the aforementioned assays with individual organisms (three experiments, 10 individuals in each) to measure microbial levels and variation in L3 larvae and adult flies (Fig. S5). The fractions of L3 larvae that harbored no *A. pasteurianus* were comparable between *Wolbachia-*free and -infected flies (20% versus 17%, respectively, *P* = 0.741) (Fig. S5A). There was also no *Wolbachia*-dependent effect on the level of either *A. pasteurianus* or *L. plantarum* relative to that in the host (Fig. S5B and C). The bacterial levels on the food were similar in *Wolbachia-*free and -infected bottles at both the L3 larval and 10-day-old adult stages (Fig. S6). However, the levels of bacteria in this genotype are, notably, at least an order of magnitude lower than in the genotype where the presence of *Wolbachia* bacteria affected their levels, even though similar amounts of bacteria were seeded to produce the gnotobiotic organisms. These results show that the effect of *Wolbachia* bacteria on the gut microbiota can be sensitive to the host genotype. Further assessment of a wide range of isogenized host genotypes will shed light on specific genetic factors that influence host-*Wolbachia* interactions that, in turn, can affect commensal microbes.

10.1128/mSphere.00287-17.5 FIG S5  The effect of *Wolbachia* infection on the microbiome is genotype dependent. *A. pasteurianus* levels in flies with a different genetic background are shown (white). (A) Proportions of individual L3 larvae with a different genetic background (white) that had no *A. pasteurianus* in the gut (*n* = 30, chi-square test; error bars show confidence intervals). (B, C) Box-and-whisker plots of levels of bacteria in L3 larvae (B) and 10-day adults (C) (*n* = 30, median values are shown next to the boxes, and *P* values obtained with the two-sided Wilcoxon rank sum test are reported). (D) Relative expression of Imd pathway signal transducer *imd*, transcription factor *Rel*, AMPs, *Nox*, and AMPs downstream of JAK/STAT signaling in both axenic and gnotobiotic L3 larval guts in the presence or absence of *Wolbachia* infection as determined by RT-qPCR. All conditions are normalized to *Wolbachia-*free gnotobiotic L3 larval guts. Bar graphs show mean values (three biological replicates of 10 larvae each), and error bars show standard deviations. Asterisks indicate statistically significant differences (*P* < 0.05, Student *t* test). Download FIG S5, TIF file, 0.8 MB. Copyright © 2017 Simhadri et al. 2017 Simhadri et al. This content is distributed under the terms of the Creative Commons Attribution 4.0 International license.

10.1128/mSphere.00287-17.6 FIG S6  Bacterial levels in the food of *w*1118 (white-eyed) flies. Levels of each bacterial species per nanogram of DNA obtained from the food, normalized to the levels in food inhabited by *Wolbachia-*free flies, are shown. Bar graphs represent the mean values of three experiments, and error bars show standard deviations. *P* values were determined by Student *t* tests. Download FIG S6, TIF file, 0.2 MB. Copyright © 2017 Simhadri et al. 2017 Simhadri et al. This content is distributed under the terms of the Creative Commons Attribution 4.0 International license.

### *Wolbachia* bacteria are present intracellularly in the gut epithelium but absent from the lumen.

We investigated the stock (upd>hPABP-Flag) where *Wolbachia* bacteria reduced the levels of *A. pasteurianus* for possible causes of this phenotype. Toward determining the mechanistic basis for this *Wolbachia*-induced microbiome differences, we characterized the distribution of *Wolbachia* bacteria and *A. pasteurianus* in the gut by fluorescent *in situ* hybridization (FISH) (see Materials and Methods).

**FIG 4:**
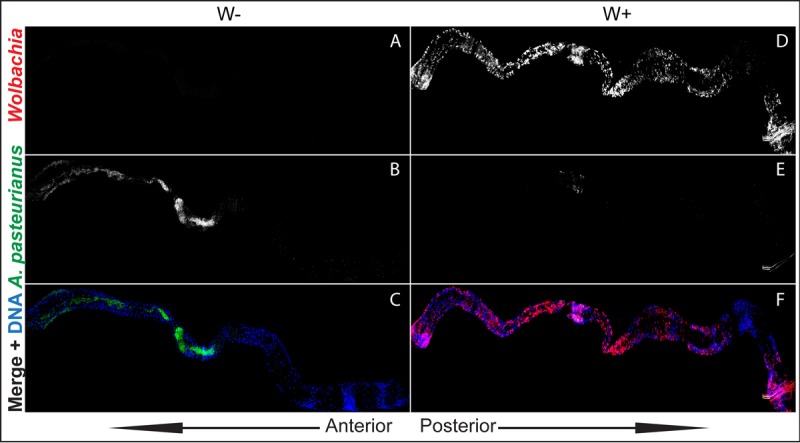
*A. pasteurianus* is absent from *Wolbachia*-infected (W+) L3 larval guts. (A to F) Composites of z-stack projections of confocal images of gnotobiotic L3 larval midguts. (A to C) *Wolbachia*-free (W−) guts. (D to F) *Wolbachia*-infected guts. (A, D) *Wolbachia* channel. (B, E) *A. pasteurianus* channel. (C, F) Merged images of *Wolbachia* (red), *A. pasteurianus* (green), and DNA (blue).

In five out of six *Wolbachia*-free guts that had *A. pasteurianus*, we observed that the bacteria were predominantly present only in the anterior midgut but not in the posterior regions or the hindgut (Fig. 4B and C). We then looked at the *Wolbachia*-infected guts to analyze if *Wolbachia* and *A. pasteurianus* bacteria were spatially exclusive. However, this was not possible, as most of the guts analyzed (three out of four) did not have any *A. pasteurianus* (Fig. 4C to E). This trend of absence of *A. pasteurianus* (*P* = 0.0325, one-tailed chi-square test) is in agreement with the previous result, where it was absent from a larger fraction of *Wolbachia*-infected than *Wolbachia*-free guts (Fig. 3D).

**FIG 5:**
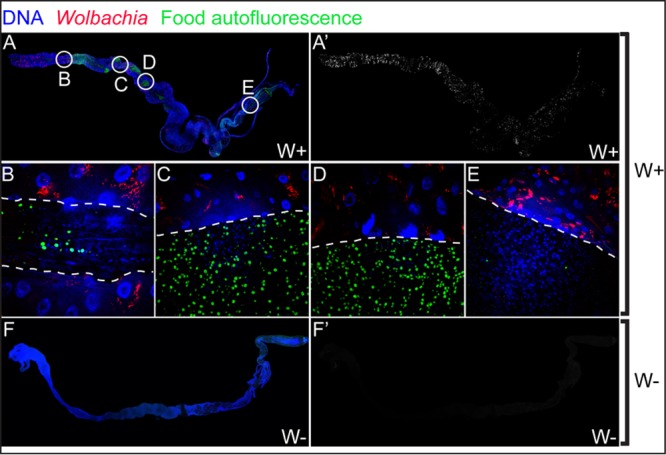
*Wolbachia* bacteria are present in gut cells but absent from the lumen. Composite confocal images of the whole midgut and hindgut of *Wolbachia*-infected (W+) flies (A) and *Wolbachia-*free (W−) flies (F) and the respective *Wolbachia* channels (A′ and F′). Magnification (×60) of the midgut (B to D) and hindgut (E) regions shows that *Wolbachia* bacteria are present intracellularly in gut cells but absent from the lumen of the gut (the lumen is marked by green autofluorescence).

Even though *Wolbachia* bacteria are primarily reproductive symbionts found in the gonads, we found them in a majority of the gut cells. *Wolbachia* bacteria did not show any preference for a specific region of the gut (Fig. 5). We also assessed the possibility of *Wolbachia* bacteria in the lumen directly affecting the gut commensal bacteria. Higher-magnification confocal images (Fig. 5B to E) showed that *Wolbachia* bacteria were present only in the gut epithelium and absent from the lumen. *Wolbachia* bacteria do not occupy the same niche as the gut microbiome, in agreement with the previously reported absence of *Wolbachia* bacteria from fecal matter (23).

### The presence of *Wolbachia* bacteria in flies does not alter the gut immune response.

**FIG 6:**
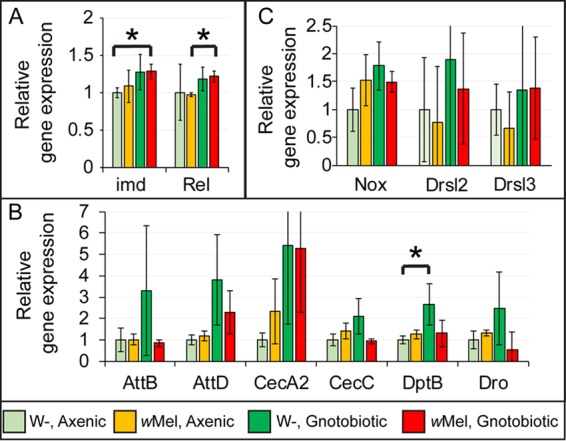
*Wolbachia* bacterial infection does not affect the expression of Imd pathway components and ROS-producing oxidases. The relative expression of Imd pathway signal transducer *imd* (A), the transcription factor *Rel* (A), AMPs (B), *Nox* (C), and AMPs downstream of JAK/STAT signaling in both axenic and gnotobiotic L3 larval guts in the presence or absence of *Wolbachia* infection was determined by RT-qPCR. All conditions are normalized to *Wolbachia-*free (W−) axenic L3 larval guts. Bar graphs show mean values (three biological replicates of 10 larvae each), and error bars show standard deviations. Asterisks indicate statistically significant differences (*P* < 0.05, Student *t* test).

Since direct interaction between *Wolbachia* bacteria and gut microbes is unlikely, we hypothesized that the presence of *Wolbachia* bacteria in flies might elicit a gut-specific immune response that could alter the composition of the gut microbiota. To examine this possibility, we first generated axenic organisms that were either *Wolbachia*-free or infected in triplicate. Since the greatest difference in the composition of the microbiome was observed at the L3 larval stage, we isolated whole guts of 10 axenic L3 larvae for each replicate, extracted whole RNA, and performed reverse transcription (RT)-qPCRs of several immunity related genes. Two major signaling pathways, Toll and Imd, control *Drosophila* systemic immunity. However, in the *Drosophila* midgut, only Imd signaling induces an immune response (15, 24). We first tested for the expression of the immune deficiency (*imd*) and Relish (*Rel*) genes, which are the key components of signal transduction in the major immunity pathway in the gut tissue, and found that there were no significant differences due to the presence of *Wolbachia* bacteria (Fig. 6A). Since antimicrobial peptide (AMP) expression is a well-characterized immune response readout, we measured the expression levels of all of the AMPs downstream of the Imd pathway. The expression of *AttB*, *AttD*, *CecA2*, *CecC*, *DptB*, and *Dro* was not different between *Wolbachia-*free and -infected L3 larval gut tissues (Fig. 6B). Other AMPs downstream of Imd, such as *Atta*, *AttC*, *CecA1*, *CecB*, and *Dpt*, were either not expressed or expressed at very low levels. These results indicate that under the axenic conditions used, *Wolbachia* bacteria alone do not activate Imd signaling.

To quantify the expression of Imd pathway genes in the presence of gut bacteria, we generated gnotobiotic larvae by seeding germfree larvae with *A. pasteurianus* and *L. plantarum* (see Materials and Methods). Compared to those in axenic larvae, the expression levels of *imd* and *Rel* were moderately upregulated in the presence of gut microbes. Importantly, this upregulation was independent of *Wolbachia* infection status. In gnotobiotic flies, *imd* and *Relish* expression is similar in *Wolbachia-*free and -infected larval guts (Fig. 6B). Regarding AMP production, again, *Atta*, *AttC*, *CecA1*, *CecB*, and *Dpt* were either not expressed or expressed at very low levels. For expressed AMPs, the introduction of commensal bacteria caused an overall increase in the levels of AMPs expressed. Because of the high variability in expression levels, only *Diptericin B* (*DptB*) was statistically significantly different. The presence of *Wolbachia* bacteria did not result in significant changes in AMP gene expression in gnotobiotic flies.

In addition, the JAK/STAT pathway is also relevant to gut innate immunity and can regulate the expression of some AMPs (25, 26). We tested *Drsl2* and *Drsl3* (also known as *dro2* and *dro3*) transcription levels in larvae with different *Wolbachia* and microbiome statuses. Again, we found no significant differences due to the presence of *Wolbachia* bacteria in axenic or gnotobiotic L3 larvae (Fig. 6C).

Another key defense mechanism in the gut is the production of reactive oxygen species (ROS). A key readout of their immune activation is upregulation of the *Nox* and *Duox* genes (27). Regardless of *Wolbachia* infection status, levels of *Duox* expression were negligible and levels of *Nox* expression were not modified (Fig. 6C). Together, these results suggest that *Wolbachia* modulation of the microbiome is independent of AMP production or *Nox* expression in the gut.

## DISCUSSION

The interaction between the resident microbes can be a key determinant of the microbial species present in a host. In the insect gut, pathogenic organisms can be excluded by resident microbes (reviewed in reference 28). The insect microbiome can also influence symbiont vertical transmission. A recent study by Hughes and collaborators showed that in *Anopheles* mosquitos, intracellular *Wolbachia* bacteria are robustly excluded from vertical transmission by *Asaia* sp., an acetic acid bacterium (17). Here, we show for the first time a reciprocal aspect of commensal microbiome bacteria and symbionts; our data indicate that *Wolbachia* bacteria in *D. melanogaster* are capable of altering the composition of resident microbes compared to that in the *Wolbachia-*free host.

We initially surveyed the composition of the resident bacterial species in a *Drosophila* stock reared in our lab—upd>hPABP-Flag—and found that *A. pasteurianus* and *L. plantarum* are the only two species in this strain of flies. This is in general agreement with reports from other groups that list *Acetobacter* and *Lactobacillus* as genera commonly associated with *Drosophila* flies reared in the lab and also wild-caught fly strains (10, 12, 29). We compared the microbial composition of another stock with the same genetic background that was not infected with *Wolbachia* bacteria. Surprisingly, the levels of *A. pasteurianus* are lower in both male and female *Wolbachia*-infected flies than in *Wolbachia-*free flies. By validating this result by PCR with species-specific primers, we also showed that the levels of *A. pasteurianus* are consistently reduced in adults and larvae.

These differences in the microbiome could be due to the presence of *Wolbachia* bacteria or could reflect an event unrelated to *Wolbachia* bacteria that is maintained across generations. Since the microbiome is transmitted vertically via fly feces to the next generation, the difference in the levels of bacteria that we observed could be due to just lower seeding from previous generations. To eliminate the effect of parental transmission of microbes to offspring, we studied the effects of *Wolbachia* bacteria on flies raised from gnotobiotic embryos. The results show that *Wolbachia* infection is sufficient to reduce the levels of *A. pasteurianus* at certain developmental stages. Specifically, the *A. pasteurianus* level is considerably lower in *Wolbachia*-infected larvae than in their *Wolbachia-*free counterparts. We show that this reduction is due to a complete lack of *A. pasteurianus* in a significant fraction of the *Wolbachia*-infected L3 larvae compared to *Wolbachia-*free larvae. Further, by using FISH to probe for *A. pasteurianus* in L3 larval guts, we observed a marked absence of *A. pasteurianus* in a majority of *Wolbachia*-infected guts but not in *Wolbachia*-free guts. Recent work with *Anopheles stephensi* mosquitos also shows variation of *Wolbachia*-induced differences in the mosquito microbiome. Directly after a blood meal, *Wolbachia*-infected mosquitos had a smaller proportion of gammaproteobacteria than *Wolbachia*-cured mosquitos did (see Fig. 2 in reference 30). A week after a blood meal, *Wolbachia*-infected mosquitos have a significantly more diverse microbiota than *Wolbachia*-free insects. However, when fed just a sugar meal or immediately after a blood meal, there are no differences between *Wolbachia-*free and -infected mosquitos (see Table 1 in reference 30). Another study of *D. melanogaster* showed that the presence of *Wolbachia* reduced the diversity of the gut microbiome (see Fig. 2B in reference 32), and also reduced the abundance of Acetobacter genus (see Table 2 in reference 32).

In contrast to the results obtained with larvae, the relative levels of *A. pasteurianus* in young gnotobiotic adult flies were not significantly different from those in *Wolbachia-*free adult flies. This could be a result of loss of the microbiome during histolysis of the larval gut during pupation. In concordance, several studies showed that newly eclosed adults have extremely low densities of resident bacteria and are recolonized by feeding (9, 31). Therefore, any *Wolbachia*-induced reduction of *A. pasteurianus* in the larval stages is lost and must be reestablished over time. This explains the large variability in the relative quantities of the gut microbes in both *Wolbachia-*free and -infected young adults. On the other hand, conventionally reared *Wolbachia*-infected adults have greatly reduced levels of *A. pasteurianus* since the bacteria are not externally introduced into the food. In the case of conventionally reared flies, the only source of *A. pasteurianus* bacteria available to offspring is adults, and from our data, they are not detected or greatly reduced.

We used another genotype, *w*1118, to address whether the host genotype plays a role in this *Wolbachia*-induced microbial change. We did not detect any significant effect of *Wolbachia* bacteria on either *A. pasteurianus* or *L. plantarum* levels at the L3 larval stage or in 10-day-old adult flies. Since the levels of *A. pasteurianus* in flies of the *w*1118 genotype are significantly lower than in those of the upd>hPABP-Flag genotype, *Wolbachia*-induced effects on *A. pasteurianus*, if there are any, might be harder to detect. Further, the two genotypes used here have quite a few dissimilarities, including a balancer chromosome and the UAS-GAL4 system in upd>hPABP-Flag flies, which is absent from *w*1118 flies. Thus, the host genotype can play a role in determining the outcome of microbial populations in the gut. Findings presented here are in accordance with mounting evidence showing a complex interaction of *Wolbachia* bacteria and commensal microbial composition influenced by several variables, including host genotypes (17, 30, 32–34). A thorough future investigation of host and microbial genetic and epigenetic determinants that can alter the tolerance and carrying capacity of the host for each of the colonizing microbes across a wide range of insect species is needed. Taking this into account, we investigated the upd>hPABP-Flag genotype, in which *Wolbachia* bacteria play a role in the determination of the microbial composition, for a possible mechanism of this phenotype.

There are several possible mechanisms for modulation of the microbiome by *Wolbachia* bacteria. *A. pasteurianus* levels could be reduced because of direct competition for nutrients; however, this is unlikely since ingested nutrients are immediately available to *A. pasteurianus*, which is present in the lumen, while *Wolbachia* bacteria are intracellular. Additionally, factors derived from either *Wolbachia* bacteria or the host could inhibit *A. pasteurianus*.

Regarding *Wolbachia*-derived factors, *Wolbachia* bacteria contain a type 4 secretion system (35), which can be used to secrete factors that subvert host cell biology to favor bacterial survival and growth. However, there is no evidence of a *Wolbachia*-derived factor that could directly influence the presence of other bacteria. Such a factor, to be effective against *A. pasteurianus*, would have to be exported into the gut lumen. Since both the host genotypes tested harbor the same strain of *Wolbachia*, and yet do not produce the same microbiome composition, *Wolbachia* secreted factors are unlikely to play a role. Therefore, we favor host-derived factors as the most likely mechanism.

A second possibility is indirect inhibition of *A. pasteurianus* by *Wolbachia* bacteria via the host. The host immune system, specifically AMPs, could play a role in altering the microbiome in response to *Wolbachia* infection. Previous studies of host immunity showing that *Wolbachia* bacteria upregulate the immune response were performed with nonnative hosts that were transinfected with *Wolbachia* bacteria from another host (36–39). However, similar studies performed with a host such as *D. melanogaster* natively infected with *Wolbachia* strain *w*Mel did not show any systemic upregulation of immunity (39, 40). Though both native and nonnative *Wolbachia*-infected hosts exhibit a robust antiviral response to single-stranded RNA viruses (37, 41–44), the native host (*Drosophila*, in this case) did not show any antibacterial activity when infected with pathogenic strains of bacteria via injury (40, 45). No previous study addressed *Wolbachia*-induced antimicrobial effects on commensal gut microbes or *Wolbachia* putative immune regulation specifically in the digestive tract. Regulation of the immune response in the fly gut differs in several aspects of systemic immunity. It is possible that *Wolbachia* infection could be generating an intestine-specific immune response acting to destabilize the microbiome. We tested this hypothesis in both axenic and gnotobiotic organisms that were either *Wolbachia* free or infected. There were no significant changes in the expression of AMPs of the Imd or JAK/STAT pathway between *Wolbachia*-infected and *Wolbachia*-free L3 larval gut tissues under both axenic and gnotobiotic conditions.

Finally, ROS could also play a role in modulation of the microbiome in response to infection with intracellular bacteria like those of the genus *Wolbachia*. There are several papers indicating that *Wolbachia* bacteria upregulate ROS in their insect host (46–50). Most of the measurements were done by using the whole organism. The amplitude of the response varies according to the tissue; data from naive hosts suggest that while changes in the major ROS effector genes *Nox* and *Duox* can be significantly affected by *Wolbachia* bacteria at the systemic level, in the gut, there are no significant differences (47). In agreement, we also did not observe *Wolbachia*-driven changes in the levels of *Nox* or *Duox* expression in the fly gut. These results suggest that the mechanism of *Wolbachia* modulation of the microbiome does not operate through simple changes in the expression of genes for the two major classes of key effectors of gut immunity, the genes for AMPs and Nox. These findings highlight the complexity of the interaction of *Wolbachia* bacteria with their hosts.

Our results do not rule out the possibility of the involvement of these pathways at the posttranscriptional level. Besides changes at the translational and posttranslational control levels, these pathways affect gut physiology beyond a simple immune response (51–53). For instance, it is well established that ROS (53) and JAK/STAT signaling (51) affects stem cell proliferation and regulates turnover of the gut epithelium. Other physiological aspects, such as the gut pH, have been shown to be affected by commensal microbiota (52). Differences in the gut pH due to the presence of *Wolbachia* bacteria could selectively influence the presence of *A. pasteurianus* and *L. plantarum*. Future studies should provide further insight into the mechanisms of *Wolbachia*-induced changes in the composition of the microbiome.

Our data show that the presence of *Wolbachia* bacteria has a significant effect on the composition of the microbiome of bacterial species in certain host genotypes under lab conditions. Though this effect might not be generalizable to every fly genotype and host species, these finding are relevant for studies investigating the phenotypic consequences of *Wolbachia* infection on the host. *Wolbachia* bacteria have been shown to be capable of altering many phenotypes in insects, such as fecundity (6, 7), insulin signaling and metabolism (7), immunity and resistance to pathogens (36–39, 41, 42, 54, 55), stem cell activity (6), and life span (56). With growing evidence that resident gut microbes are also capable of altering many of these phenotypes (16, 57–62), it is important to delineate the relative contributions of each of the bacteria to the phenotypic changes seen.

Modulation of the microbiome by *Wolbachia* bacteria may have dramatic effects on host fitness. For example, in terms of immunity, it is known that a certain composition of the microbiome confers protection against pathogens on several organisms, from plants to humans (63, 64). In *Drosophila*, it has been reported that higher relative levels of *L. plantarum* promote protection against *Serratia marcescens* and *Pseudomonas aeruginosa*, two known *Drosophila* pathogens that also cause opportunistic infections in humans (31). These findings raise the possibility that *Wolbachia* bacteria change the host defense indirectly by affecting the composition of the microbiome. Altered immune competence can play a key role in the survival of host populations in nature.

The results shown here are also relevant for the development of bacterium-based approaches to vector control. Several studies have shown that gut and *Wolbachia* bacteria inhibit the presence of human pathogens in insect vectors, including *Plasmodium falciparum* and the dengue, West Nile, and chikungunya viruses (reviewed in references 8, 28, 36–39, 41, 42, 54, and 55). Therefore, it is important to understand the interactions of *Wolbachia* bacteria with other bacteria that inhibit disease transmission in order to determine the effects of synergistic or antagonistic interactions on vector control.

## MATERIALS AND METHODS

### Fly strains, *Wolbachia* typing, and husbandry.

The *D. mauritiana* fly stocks used in this study were from Fast et. al. (6). The *D. melanogaster* fly stocks used in this study were upd-Gal4; Cyo/Sco; P(UAS-hPF)B (upd>hPABP-Flag in short) and white mutant (w^1118^). upd>hPABP-Flag fly stocks with and without *Wolbachia* bacteria have similar genetic backgrounds through introgression backcrosses, as previously described (according to reference 65), and white mutant (*w*) flies with and without *Wolbachia* bacteria were a generous gift from Luis Teixeira (66). The fly food used was of the typical cornmeal-yeast-molasses-agar type (agar at 9.7 g/liter, cornmeal at 65.7 g/liter, yeast at 27 g/liter, molasses at 65.7 ml/liter) with preservatives (propionic acid at 6.9 ml/liter and tegosept at 4 ml/liter). All stocks and experiments were maintained in a 25°C incubator with 60% humidity.

*Wolbachia* typing was performed by PCR against VNTRs 141 and 105 (67).

### Egg collection and grape juice agar plates.

Fifteen newly eclosed *Wolbachia-*free and -infected *D. mauritiana* flies were collected and kept in bottles containing yeasted grape juice agar plates. Bottles were maintained at 25°C with 60% humidity. Plates were changed every 24 h and stored at 4°C.

### DNA extraction from flies and food.

Genomic DNA was isolated by using a modified form of the protocol for the Qiagen DNeasy Blood and Tissue kit (catalog no. 69506). We surface sterilized flies by vortexing them with a 50% household bleach solution (4% sodium hypochlorite) for 5 min and washing them three times with sterile water. Effective removal of external bacteria was confirmed as previously described, with modifications (10, 62). The efficiency of this procedure was confirmed by PCR assay of the water from the final wash with universal 16S rRNA gene primers. Flies were then homogenized in 200 µl of lysis buffer (20 mM Tris [pH 8.0], 2 mM EDTA, 1.2% Triton X-100) with lysozyme (MP Biomedicals, catalog no. 210083401) at 20 mg/ml and incubated for 90 min at 37°C. A 200-µl volume of AL buffer (Qiagen Blood and Tissue kit) and 20 µl of proteinase K were then added to the mixture, which was incubated further for 90 min at 56°C. Subsequent extraction with the columns was performed as recommended by the kit’s manufacturer.

To isolate DNA from the food, 50 to 100 mg of food was collected from the bottles and the protocol described above without bleach treatment was used.

### Elimination of *Wolbachia* 16S rRNA gene sequences from samples.

Total genomic DNA was extracted and digested with the NEB BstZ17I restriction enzyme (catalog no. R0594S) for 1 h at 37°C, followed by 10 min at 65°C to prevent amplification of the *Wolbachia* V1 and V2 regions of the 16S rRNA gene prior to high-throughput sequencing of the bacterial 16S rRNA gene on the MiSeq platform.

### 16S rRNA gene library preparation and sequencing.

A 500-ng portion of BstZ17I-digested total genomic DNA was used per sample for each library. All PCRs were performed with Platinum *Taq* DNA Polymerase High Fidelity from Life Technologies, Inc. (catalog no. 10790-020), and appropriate primers (Table S1). 16S rRNA gene amplicons from PCRs were separated by agarose gel electrophoresis and subsequently extracted with the Qiagen QIAquick gel extraction kit (catalog no. 28706). Kappa Biosystems DNA standards (catalog no. KK4903) were used for calibration of the DNA concentration used for sequencing by qPCR with Illumina adaptors (Table S1). qPCRs were performed with SYBR GreenER qPCR SuperMix Universal from Life Technologies, Inc. (catalog no. 11790-01k). Sequencing was performed with the Illumina MiSeq platform by using 250-bp paired-end reads. Analysis of the reads was performed with the QIIME 1.8.0 package. Default parameters were used for the analysis, and the Greengenes database was used to assign taxonomy.

### Isolation and identification of bacteria from flies.

Twenty flies were homogenized in 500 µl of MRS medium (catalog no. 288130; BD), fly debris was removed by brief centrifugation in a tabletop centrifuge, and 20 µl of the supernatant was plated on MRS agar. The plates were incubated at either 37 or 30°C. Sixteen colonies were sampled, and colony PCR was performed with the universal bacterial 16S rRNA gene primers. The PCR amplicons were Sanger sequenced with both forward and reverse universal 16S rRNA gene primers.

### Bacterial cultures.

All bacterial cultures were grown in MRS liquid medium or agar (catalog no. 288130; BD). *L. plantarum* cultures were grown at 37°C, and *A. pasteurianus* cultures were grown at 30°C.

### Bacterial whole-genome sequencing.

DNA was extracted from overnight cultures of *L. plantarum* and *A. pasteurianus* by a modified version of the protocol for the Qiagen blood and tissue kit as described above. DNA was sheared with Covaris spin tubes (catalog no. 520079). Genome libraries were prepared in accordance with the PacBio Template Preparation and Sequencing Guide selecting for approximately 10-kb genome fragments. DNA quality and size were confirmed on a Bioanalyzer, followed by sequencing with a PacBio RS II sequencer. Raw reads were assembled *de novo* by SMRT analysis software. Manual curation and closing of the genome were done by NCBI alignment. The *A. pasteurianus* chromosome (3.12 Mb) and plasmid (140 kb) were annotated by using a database of closed *Acetobacter* strains (CP012111 and NC_013209). The *L. plantarum* (3.32 Mb) genome was annotated by rapid annotations using subsystems technology (68, 69).

### Gnotobiotic flies.

Gnotobiotic flies were generated by exposing sterile embryos to 1,000 CFU of the required bacteria on sterile fly food. Embryos were sterilized by three washes in 50% bleach (Clorox diluted in sterile water to a final concentration of approximately 4% sodium hypochlorite) for 2 min per wash. Subsequently, the dechorionated sterile embryos were washed three times with a sterile Triton salt solution (4 g/liter NaCl, 300 µl/liter Triton X-100) to prevent sticking to surfaces. After removal of all the Triton salt solution, an equal volume of fresh Triton salt solution was added to the embryos. A 10-µl volume of this mixture was added to autoclave-sterilized fly food. Sterilized fly food was prepared similarly to regular fly food without preservatives after autoclaving. A correlation of the optical density (OD) at 600 nm and the CFU count was generated for each species; *L. plantarum* was 1.11 × 10^9^ CFU/ml/OD unit, and *A. pasteurianus* was 2.6 × 10^8^ CFU/ml/OD unit. By using appropriate dilutions of log-phase cultures whose ODs were measured, 1,000 CFU each of *A. pasteurianus* and *L. plantarum* were added to sterile hatching L1 larvae. L3 larvae and adult flies were collected under sterile conditions, and their DNA was extracted after surface sterilization with 50% bleach.

### *Wolbachia* and *A. pasteurianus* immunocytochemistry.

*Wolbachia in situ* localization in the gut was determined by FISH as previously described (65). *A. pasteurianus* probes were designed and tested for specificity. The probe used in this study was 5′ 6-carboxyfluorescein--AGAGTGCCCAGCCCAACCTGA from IDT DNA. Both *Wolbachia* and *A. pasteurianus* probes were used at 1 ng/µl. To perform FISH of gut contents, the fly food was modified by replacing yeast with yeast extract and sugars to eliminate the autofluorescence of yeast. The composition of the modified fly food was dextrose at 50 g/liter, sucrose at 25 g/liter, yeast extract at 15 g/liter, cornmeal at 60 g/liter, agar at 6.5 g/liter, tryptone at 30 g/liter, and molasses at 65 g/liter. L3 larval guts were dissected and fixed in 4% paraformaldehyde in phosphate-buffered saline (PBS) for 1 h and then in 50% ethanol with PBS for 30 min at −20°C. Hybridization was performed as previously described (65). Image acquisition was performed with an Olympus FluoView 1000 confocal microscope. Full-gut images were assembled with Microsoft Image Composite Editor and the MosaicJ package in FIJI from individual images collected at ×40 (for Fig. 4) or ×60 (for Fig. 5) magnification. Images were processed with Photoshop to eliminate pixels outside the gut and equalize channel intensity within the same composite image.

### qPCR.

qPCRs were performed with SYBR GreenER qPCR SuperMix Universal from Life Technologies, Inc. (catalog no. 11790-01k). BstZ17I was used to digest 200 ng of total genomic DNA for 1 h at 37°C, followed by 10 min at 65°C. A 5-ng sample of the digested DNA was amplified with the following species-specific primers: *A. pasteurianus*, forward primer 5′ GCACCCTCATGGTACCGAGC 3′ and reverse primer 5′ ACCAGCAGGGCGATGGTTTC 3′; *L. plantarum*, forward primer 5′ ACGTTAGGGCTACTCGGCCA 3′ and reverse primer 5′ GCCTTCGCCGACCCCAATTA 3′. Universal 16S rRNA gene primers or *D. melanogaster* 14-3-3 gene forward primer 5′ CATGAACGATCTGCCACCAAC 3′ and reverse primer 5′ CTCTTCGCTCAGTGTATCCAAC 3′ were used for normalization.

### RT-qPCRs for transcriptional profiling.

Ten guts of axenic or gnotobiotic L3 larvae were dissected in Grace’s insect medium (Lonza catalog no. 04-457F), and the RNA was immediately extracted with the Qiagen miRNeasy minikit (catalog no. 217004) in accordance with the manufacturer’s protocol. For quantification of the mRNA levels of the genes in the Imd pathway, the EXPRESS one-Step SYBR GreenER kit with premixed ROX from Life Technologies, Inc., was used (catalog no. 1179001k). Twenty nanograms of RNA was used as the input for each reaction, and the conditions used were those recommended by the kit’s manufacturer.

### Accession number(s).

The accession numbers of the BioProjects containing the raw reads from this study in the NCBI database are PRJNA381361 (16S rRNA gene raw reads) and PRJNA384998 (whole-genome sequencing of *A. pasteurianus* and *L. plantarum*).
