# Supplementary figures and images for: The Gut Commensal Microbiome of Drosophila melanogaster Is Modified by the Endosymbiont Wolbachia
Source: mSphere. 2017 Sep 13;2(5):e00287-17. doi: 10.1128/mSphere.00287-17 (PMC5597968; doi:10.1128/mSphere.00287-17)

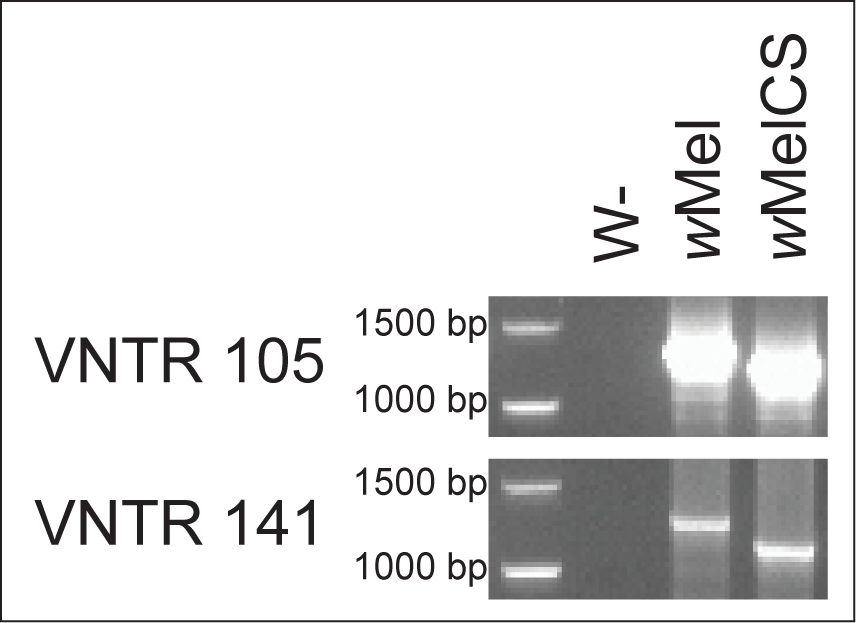

Supplement: FIG S1 [file sph004172337sf1.tif]

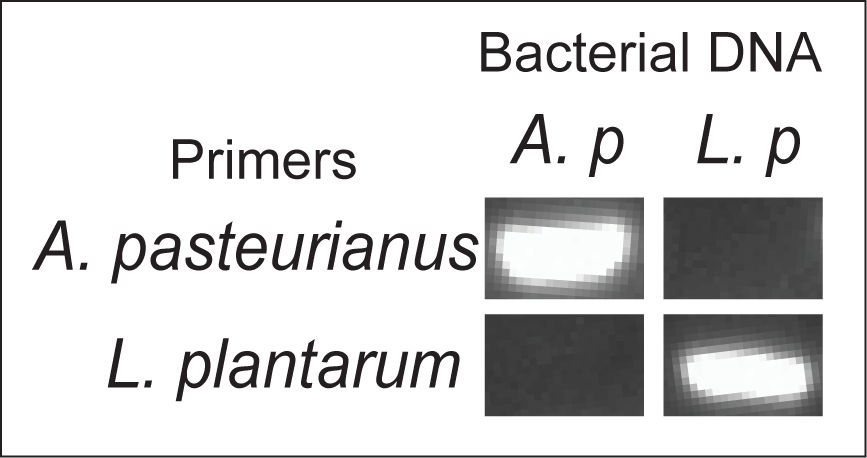

Supplement: FIG S2 [file sph004172337sf2.tif]

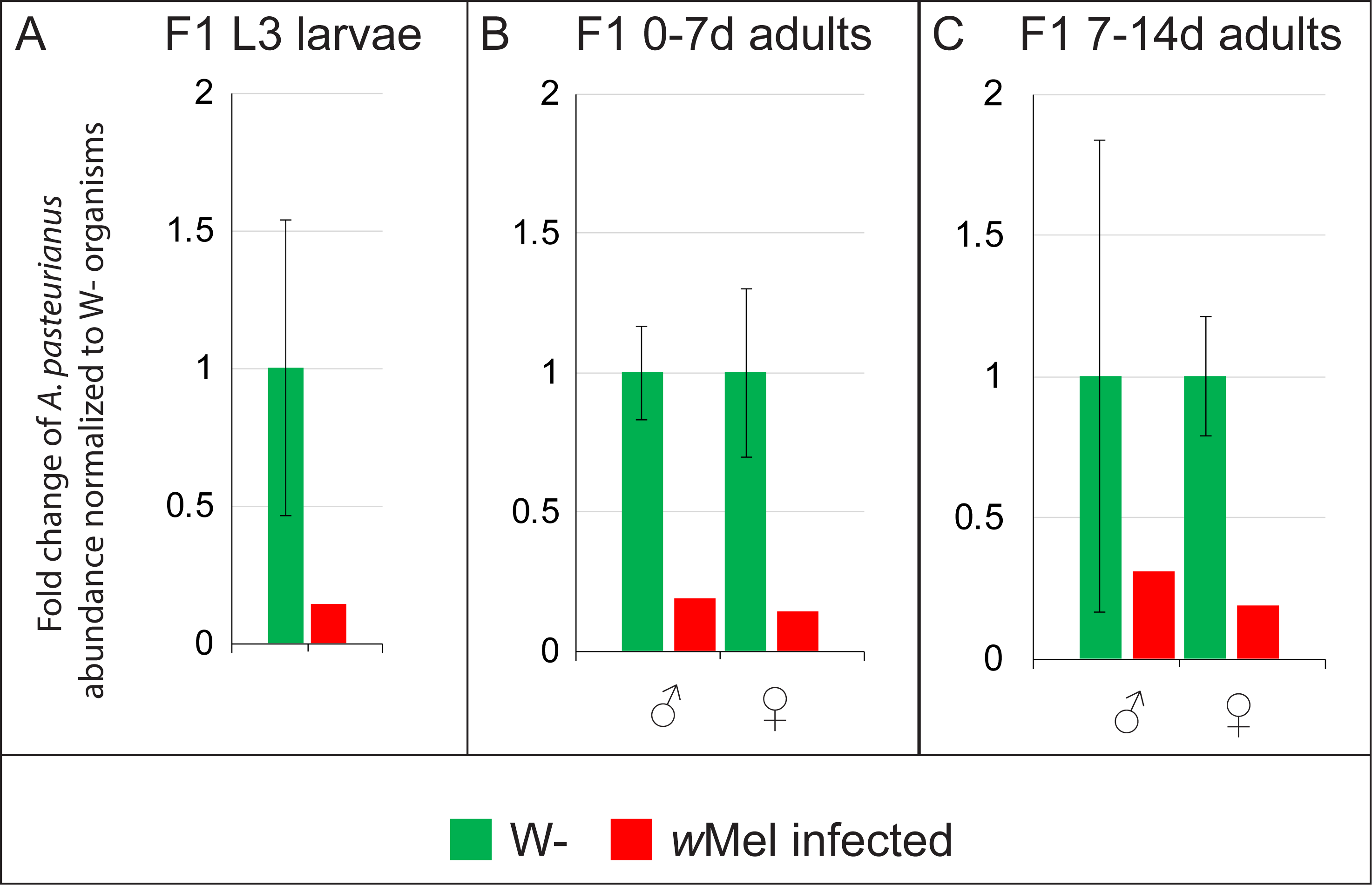

Supplement: FIG S3 [file sph004172337sf3.tif]

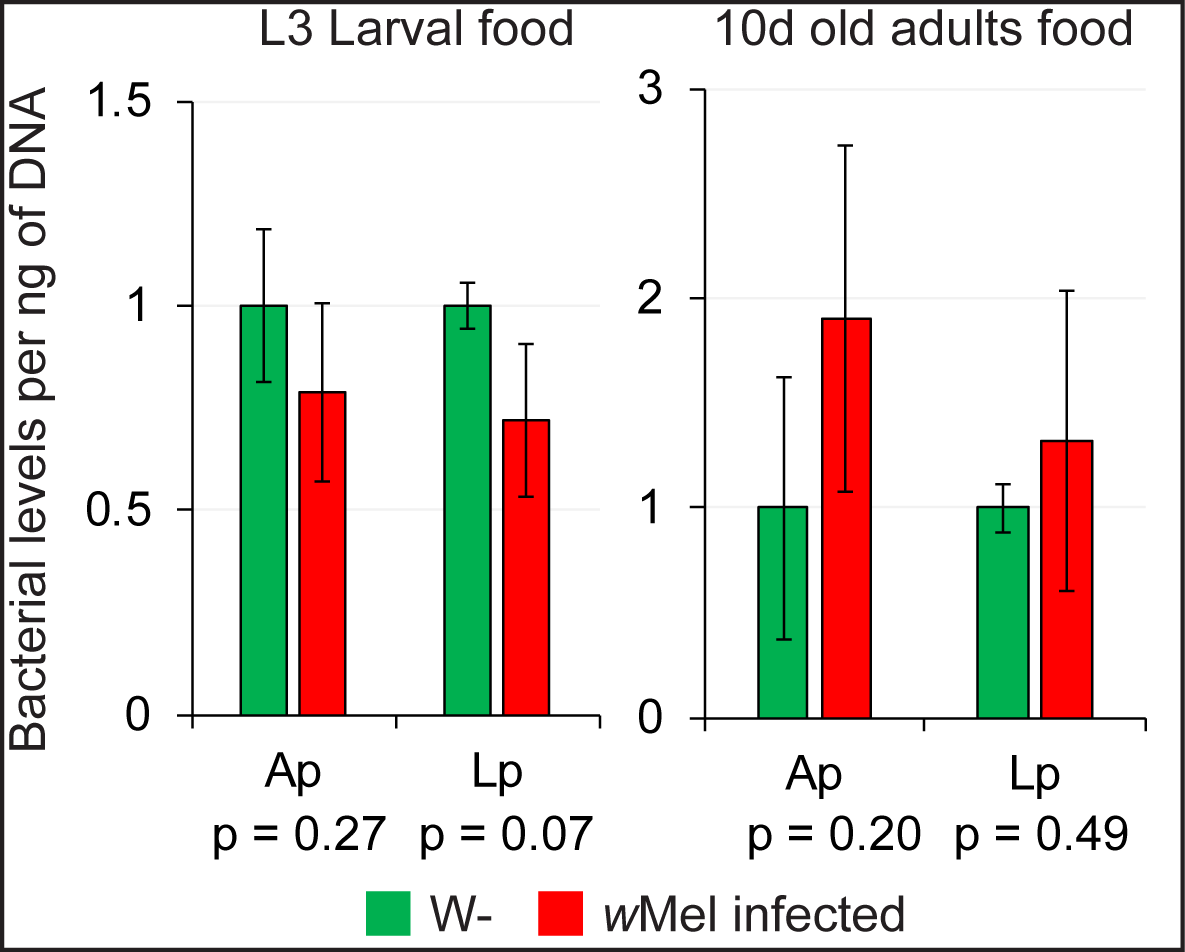

Supplement: FIG S4 [file sph004172337sf4.tif]

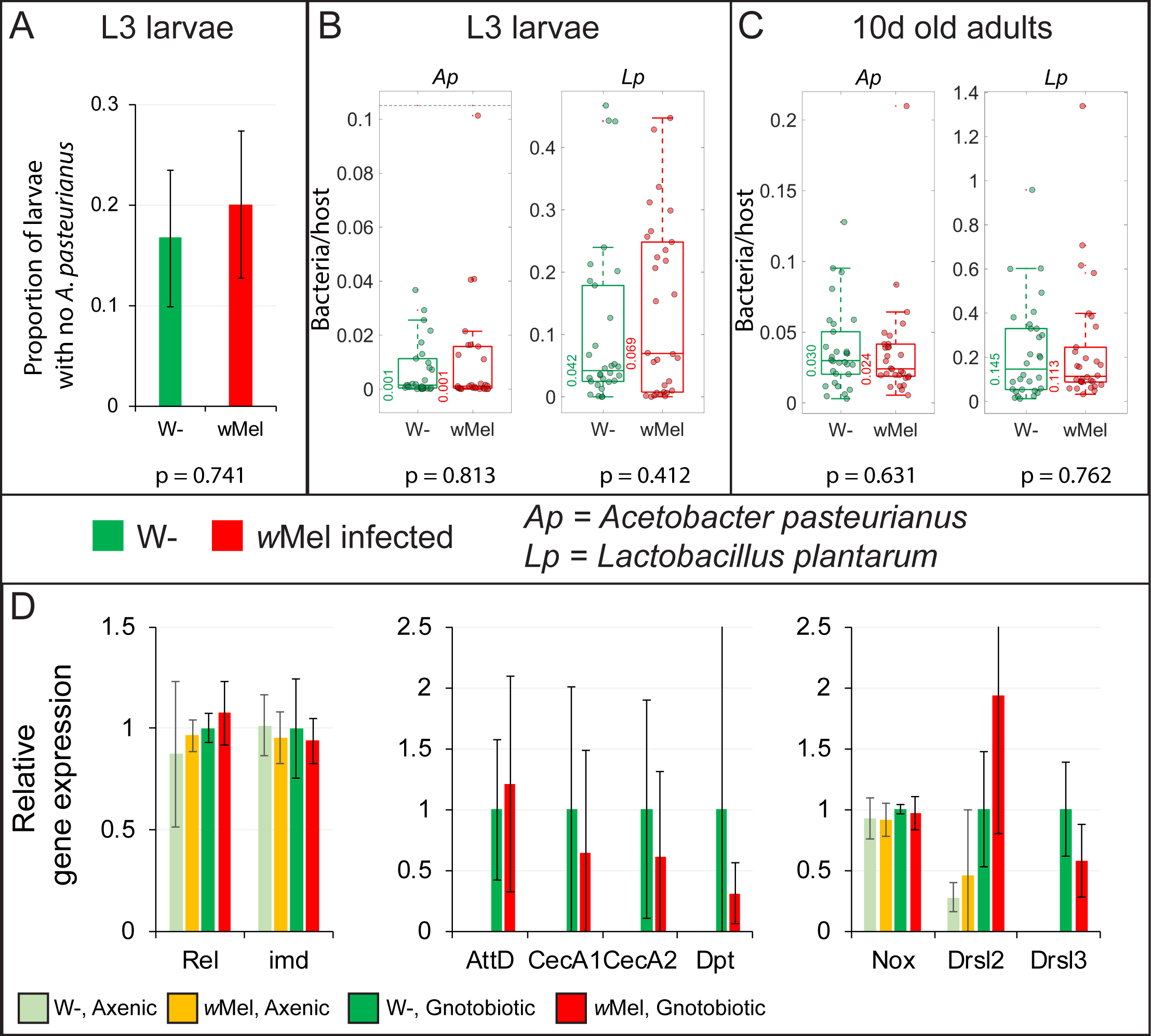

Supplement: FIG S5 [file sph004172337sf5.tif]

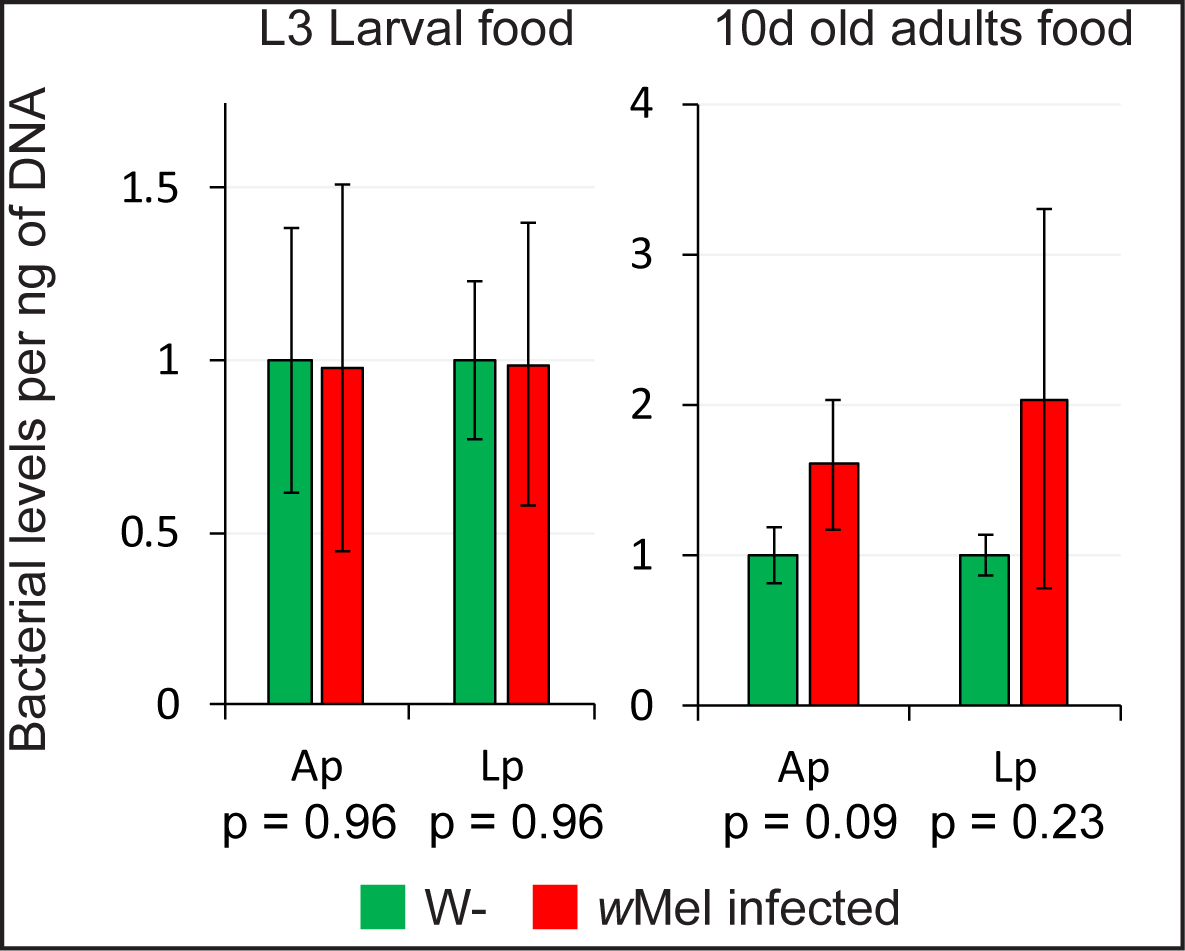

Supplement: FIG S6 [file sph004172337sf6.tif]
