# Supplementary material for: The Gut Commensal Microbiome of Drosophila melanogaster Is Modified by the Endosymbiont Wolbachia
Source: mSphere. 2017 Sep 13;2(5):e00287-17. doi: 10.1128/mSphere.00287-17 (PMC5597968; doi:10.1128/mSphere.00287-17)
Supplement: TABLE S1 [file sph004172337st7.docx]

|  | BstZ17I digested prior to PCR | Number of reads | Fraction of *Wolbachia* reads | Number of *Wolbachia* reads |
| --- | --- | --- | --- | --- |
| W- Female | Yes | 114263 | 0.000255493101686 | 29 |
| W- Male | Yes | 38980 | 0.00164928708236 | 64 |
| *w*Mel Female | Yes | 98656 | 0.00304534321666 | 300 |
| *w*Mel Male | Yes | 34940 | 0.00341541575308 | 119 |
| W- Female | No | 42049 | 0.000252003 | 11 |
| W- Male | No | 45406 | 0.0004679 | 21 |
| *w*Mel Female | No | 53757 | 0.697802742 | 35712 |
| *w*Mel Male | No | 30173 | 0.910362365 | 27468 |
