# Supplementary material for: The Gut Commensal Microbiome of Drosophila melanogaster Is Modified by the Endosymbiont Wolbachia
Source: mSphere. 2017 Sep 13;2(5):e00287-17. doi: 10.1128/mSphere.00287-17 (PMC5597968; doi:10.1128/mSphere.00287-17)
Supplement: TABLE S2 [file sph004172337st8.docx]

| Primer name | Sequence |
| --- | --- |
| Bacterial 16S 27F | AGAGTTTGATCMTGGCTCAG |
| Bacterial 16S 338R | TGCTGCCTCCCGTAGGAGT |
| Bacterial 16S 27F with Illumina adaptor | AATGATACGGCGACCACCGAGATCTACACGGCTACTATGGTAATTCTAGAGTTTGATCMTGGCTCAG |
| Bacterial 16S 338R with Illumina adaptor for W- female | CAAGCAGAAGACGGCATACGAGATTAGCTTAGTCAGTCAGATTGCTGCCTCCCGTAGGAGT |
| Bacterial 16S 338R with Illumina adaptor for W- male | CAAGCAGAAGACGGCATACGAGATCAGATCAGTCAGTCAGATTGCTGCCTCCCGTAGGAGT |
| Bacterial 16S 338R with Illumina adaptor for wMel female | CAAGCAGAAGACGGCATACGAGATGGCTACAGTCAGTCAGATTGCTGCCTCCCGTAGGAGT |
| Bacterial 16S 338R with Illumina adaptor for wMel male | CAAGCAGAAGACGGCATACGAGATACTTGAAGTCAGTCAGATTGCTGCCTCCCGTAGGAGT |
| P5 adaptor primer for quantification of amplified DNA | AATGATACGGCGACCACCGAGAT |
| P7 adaptor primer for quantification of amplified DNA | CAAGCAGAAGACGGCATACGA |
