# Supplementary material for: The Gut Commensal Microbiome of Drosophila melanogaster Is Modified by the Endosymbiont Wolbachia
Source: mSphere. 2017 Sep 13;2(5):e00287-17. doi: 10.1128/mSphere.00287-17 (PMC5597968; doi:10.1128/mSphere.00287-17)
Supplement: TABLE S3 [file sph004172337st9.docx]

| Family | Female W- | Male W- | wMel Female | wMel Male |
| --- | --- | --- | --- | --- |
| *Acetobacter*aceae | 62.4371752 | 40.99654 | 3.2548224 | 17.33446 |
| Lactobacillaceae | 37.5351393 | 58.87557 | 96.7308792 | 82.64438 |
| Unassigned | 0.0170372 | 0.015987 | 0 | 0 |
| Propionibacteriaceae | 0.00426 | 0 | 0 | 0.00423 |
| Erysipelotrichaceae | 0.00213 | 0.00533 | 0 | 0 |
| Pseudomonadaceae | 0.00213 | 0.026645 | 0.0013 | 0.00423 |
| Xanthomonadaceae | 0.00213 | 0.010658 | 0.0026 | 0.00423 |
| Corynebacteriaceae | 0 | 0.010658 | 0 | 0 |
| Bacillaceae | 0 | 0.010658 | 0.0026 | 0.00423 |
| OP11-4* | 0 | 0 | 0.0026 | 0 |
| Methylobacteriaceae | 0 | 0.031974 | 0.0039 | 0 |
| Sphingomonadaceae | 0 | 0.00533 | 0.0013 | 0.00423 |
| Burkholderiaceae | 0 | 0.010658 | 0 | 0 |
| *Acetobacter*aceae + Lactobacillaceae | 99.9723145 | 99.8721 | 99.9857016 | 99.97884 |
| Total number of reads | 114234 | 38916 | 98356 | 34821 |
| Number of *Acetobacter*aceae + Lactobacillaceae reads | 114202 | 38866 | 98342 | 34814 |
| * Name of the order. Family level identity was unavailable | | | | |
